# Seroprevalence and Risk Factors of *Toxoplasma gondii* Infection in Buffalo (*Bubalus bubalis*) from Sohag, Egypt

**DOI:** 10.3390/vetsci13020166

**Published:** 2026-02-07

**Authors:** Alsagher O. Ali, Wael Qossa, Fatma A. Khalifa, Caroline F. Frey, Ragab M. Fereig

**Affiliations:** 1 Division of Infectious Diseases, Department of Animal Medicine, Faculty of Veterinary Medicine, Qena University, Qena 83523, Egyptfa.fatma@vet.svu.edu.eg (F.A.K.); 2 Veterinary Medicine Directorate, Sohag 82749, Egypt; wael23240@gmail.com; 3 Institute of Parasitology, Department of Infectious Diseases and Pathobiology, Vetsuisse-Faculty, University of Bern, Länggassstrasse 122, CH-3012 Bern, Switzerland; 4 Division of Internal Medicine, Department of Animal Medicine, Faculty of Veterinary Medicine, Qena University, Qena 83523, Egypt; 5 Department of Veterinary Medicine, College of Agriculture and Veterinary Medicine, United Arab Emirates University, Al-Ain P.O. Box 15551, United Arab Emirates

**Keywords:** *Toxoplasma gondii*, antibodies, ELISA, Sohag, buffalo

## Abstract

Toxoplasmosis is a globally prevalent protozoan parasitic disease of livestock, among others, with significant zoonotic potential. This study aimed to assess the seroprevalence of *Toxoplasma gondii* antibodies in serum samples from water buffaloes (*Bubalus bubalis*) in Sohag governorate, Upper Egypt. In addition, several factors related to animals, management, and environment were analyzed to identify the risk factors for *T. gondii* infection. A cross-sectional epidemiological approach was employed, with samples collected from various locations across the region and tested using a commercially available indirect enzyme-linked immunosorbent assay (iELISA). Animal and environmental factors were evaluated to identify potential risk factors for the disease. The overall seroprevalence of *T. gondii* among the tested buffaloes was 7% (24/342). Seasonal variations were observed, with the highest seropositivity recorded in spring (10.7%; 11/103), followed by autumn (8%; 2/25), summer (5.6%; 7/125), and winter (2.2%; 2/89). High seropositivity was detected in aborted females, at 19% (4/21), and in repeated breeders, at 10.5% (4/38), in relation to buffaloes showing anestrus (no positive cases), although the differences were not statistically significant. Our findings suggest that *T. gondii* is endemic in Sohag, with water buffaloes serving as sentinel animals for the disease. The spring season appears to be a risk factor for infection. Further studies are needed to assess the potential risk to humans, particularly regarding the consumption of raw or undercooked buffalo meat infected with *T. gondii*.

## Simple Summary

Toxoplasmosis in buffaloes represents an important One Health concern due to its combined economic, environmental, and public health implications. *Toxoplasma gondii*, the causative agent of toxoplasmosis, can induce severe clinical consequences in infected animals and humans. In Egypt, water buffaloes are used as a major source of milk and meat production. This study revealed the existence of *T. gondii* antibodies in 7% of the tested buffaloes from Sohag governorate, southern Egypt. We also found the season was a predisposing factor for seropositivity for *T. gondii*, as the highest seropositivity was recorded in spring (10.7%). *T. gondii* infection in buffaloes can pose a health risk to people—infections could be passed on by consuming raw milk or meat. This study presents novel and valuable information on the seroprevalence of *T. gondii* in Sohag, southern Egypt. These findings contribute to regional, data-driven surveillance efforts and underscore the value of buffaloes as sentinel animals for environmental contamination. Additionally, our study contributes to the regional surveillance of *T. gondii* as a zoonotic pathogen and minimizes its risks in the veterinary and public health sectors.

## 1. Introduction

Farmers rely on buffalo for their milk, meat, and long productive life [1]. Toxoplasmosis, caused by *Toxoplasma gondii*, is a widespread protozoan parasitic disease. *T. gondii* is highly adaptive and can persist long-term in tissue cysts within hosts. Felids are the definitive hosts, while various animals and humans serve as intermediate hosts [2]. Anti-*T. gondii* antibodies have been recorded in all countries and regions of the world and in most animal species [3]. Transmission occurs primarily through ingestion of contaminated food or water containing sporulated oocysts, meat containing tissue cysts, and less frequently via tachyzoites crossing the placenta, causing congenital toxoplasmosis. Approximately one-third of the global human population [2] and a quarter of livestock and poultry are infected [4].

*T. gondii* was first recognized as a cause of abortion in sheep in 1957, and has since been recognized as serious risk to pregnant animals, potentially causing abortion, mummification, stillbirth, or embryonic death. *T. gondii* is a major foodborne parasite causing toxoplasmosis, often transmitted by consuming raw/undercooked meat containing tissue cysts or unwashed produce contaminated with oocysts from cat feces [2]. It poses severe risks to pregnant women (congenital infection) and immunocompromised people [2]. In addition, *T. gondii* is a significant occupational pathogen, particularly for individuals in close contact with animals, raw meat, or contaminated soil. Prevention requires thorough cooking or freezing of meat, washing produce, and pasteurization of milk [2,5]. The only available vaccine, Toxovax^®^ (Intervet), reduces *T. gondii*-induced abortion in sheep [6].

In water buffalo, *T. gondii* has been linked to longer calving-to-conception intervals due to early embryonic death and resorption [7]. *T. gondii* in buffalo poses a potential public health risk as a food-borne pathogen through the consumption of infected meat (cysts) [8] and milk (tachyzoites) [9]. The serological prevalence of toxoplasmosis in buffalo ranges from 0% to 87.79% [10], with regional variations: 0% in Australia, 13.43% in Asia, 20% in Europe, 28.42% in North America, 10.22% in South America, and 21.15% in Africa. The weighted global prevalence is estimated at 22.26% [11].

In Egypt, *T. gondii* antibodies have been detected in various animal species, with seroprevalence rates of 38.7% in sheep, 28.7% in goats, 23.6% in cattle, 22.6% in donkeys [12], and 34.4% in buffaloes [13]. In Sohag, the overall seroprevalence in domestic ruminants (cattle, buffalo, sheep, goats) is 47.9%, with buffaloes showing a higher rate of 58.6%, indicating widespread infection in Upper Egypt [14]. In Assuit, seroprevalence varies from 20% (LAT) to 74.5% (ELISA) [15], while in Giza, it is 22.5% [16]. A recent study reported a 7.4% seroprevalence in buffaloes [17].

This study aims to assess the seroprevalence of *T. gondii* in buffaloes across different locations in Sohag and explore risk factors related to both animal and environmental factors to help inform strategies for controlling the infection. This study provided foundational epidemiological data on the presence and seasonal distribution of *T. gondii* in water buffaloes in Sohag, southern Egypt, highlighting spring as a period of elevated risk.

## 2. Materials and Methods

### 2.1. Ethical Statement

All relevant institutional, national, and/or international guidelines pertaining to the use and care of animals were followed. This study was conducted according to instructions established by the “Research Bioethic Committee- RBC” of the Faculty of Veterinary Medicine, Qena University, Qena, Egypt. The protocols were approved by the Research Code of Ethics at Qena University number “VM/SVU/25(7)-07” in accordance with the OIE guidelines for animal usage and studies. Blood samples were collected after consultation with officials and animal owners.

### 2.2. Animal Population and Geographic Location (Study Area)

**Figure 1:**
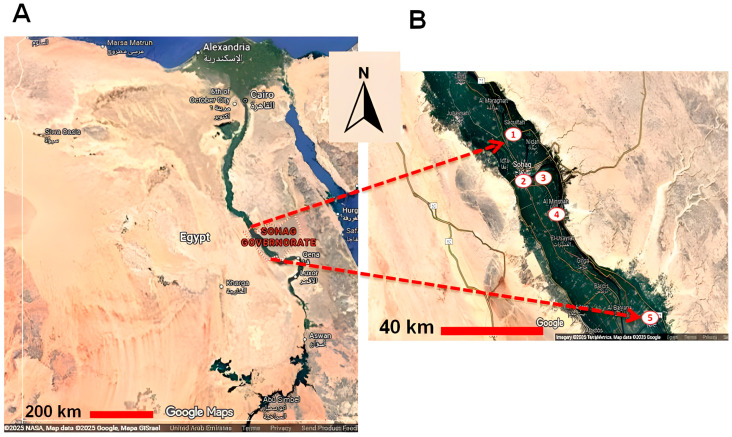
A landscape map of Egypt illustrating the cities used for sample collection in Sohag governorate. The location of Sohag governorate in upper/southern Egypt (**A**), and the location of cities used for sample collection, all situated in the Nile valley: 1: Saqultah, 2: Sohag, 3: Akhmim, 4: Al Minshah, 5: Dar-Elsalam (**B**).

This cross-sectional study was performed from October 2020 to October 2022. A total of 342 buffalo blood samples were randomly collected from five cities located in the Nile valley in the Sohag governorate, southern Egypt. Sohag governorate is located in Upper Egypt and is rich in livestock. Five cities were chosen for this study (Sohag, El-Monsha, Akhmium, Saqulta, Dar-Elsalam). Buffaloes of different ages, sexes, locations, management types, and other factors were sampled (Figure 1). The availability of samples and owner cooperation determined the numbers and groups of tested animals.

### 2.3. Sample Collection and Serum Preparation

The jugular vein was used for blood collection via venipuncture utilizing a sterile disposable plain Vacutainer tube (Smilelab^®^, Stockholm, Sweden) and needle, devoid of anticoagulants. Sera were isolated from blood samples using centrifugation at 3000 rpm for 10 min. Sera were aliquoted into 1.5 mL Eppendorf tubes post-centrifugation, transported to the laboratory of Qena University (Faculty of Veterinary Medicine, Qena University, Qena, Egypt) and maintained at −20 °C until utilized for ELISA testing.

### 2.4. Epidemiological Data

Epidemiological data were obtained by a semi-structured questionnaire distributed to animal owners to collect information on potential risk factors associated with both animals and their environment. The animal-related characteristics included age (<1 year, 1 to 3 years, >3 years), sex (female or male), body weight (<100 kg, 100 to 300 kg, >300 kg), and, for adult females exclusively, reproductive problems (yes or no). Reproductive problems were defined as abortion, recurrent breeding, and anestrus. The environmental characteristics considered included geographic location (Saqultah, Sohag, Akhmim, Al Minshah, Dar-Elsalam), sampling season (spring, summer, fall, winter), presence of cats or dogs (yes or no), and management style, as either a smallholder (5–20 animals per owner) or an individual (less than 5 animals per owner). All tested water buffaloes belonged to the local breed identified as Egyptian water buffalo breed.

### 2.5. Processing and Analysis of Data

All serum samples were tested for antibodies to *T. gondii* by iELISA using a commercial ID Screen^®^ Toxoplasmosis Indirect Multi-Species test kit based on the P30 antigen of *T. gondii* (ID. Vet. Grabels, France, TOXO-MS-2P, LOT 159). Manufacturer’s instructions were followed for preparation of samples and buffers and for all testing procedures. Except for distilled water (DW), all reagents and buffers were provided in the same kit. Serum samples and controls were diluted 1:2 and added to the wells and incubated at room temperature (24 °C) for 45 min. Then, plates were washed thrice by washing buffer provided by the company after diluting 20 times by distilled water. An amount of 100 µL of conjugated antibodies (10 times diluted) was added to the wells and incubated again at RT for 30 min. After three further washes, 100 µL of substrate solution was added to the wells and they were incubated in a dark place for 15 min. Then, stop solution (100 µL) was added to all wells and the ODs of all ELISA data were read at 450 nm and quantified using an Infinite^®^ F50 ELISA reader (Tecan Group Ltd., Mannedorf, Switzerland). The ODs obtained were used to calculate the percentage of sample (S)-to-negative (N) ratio (S/N%) for each of the test samples according to the following formula: S/N (%) = OD sample/OD negative control × 100. Samples with an S/N% more than 60% were regarded as negative, those with an S/N% between 50% and 60% were regarded as doubtful, and the test was considered positive if the S/N% was less than 50%.

### 2.6. Statistical Analysis

The significance of the differences in the prevalence rates and risk factors (age, sex, weight, reproductive problems, location, season, contact with cats and dogs, and management) was analyzed using Fisher’s exact test, 95% confidence intervals (95% CI) (including a continuity correction), and odds ratios (ORs) using an online statistical website www.vassarstats.net (accessed on 22 September 2025). Additionally, *p*-values and odds ratios were confirmed with GraphPad Prism version 5 (GraphPad Software Inc., La Jolla, CA, USA) [18].

## 3. Results

### 3.1. Seroprevalence and Associated Animal-Related Risk Factors of T. gondii in Buffaloes from Sohag

The overall seroprevalence to *T. gondii* in water buffalo of the Sohag governorate was 7% (24/342). Seropositivity was slightly higher in buffaloes > 3 years old (8.5%, 13/153) compared to those 1–3 years (5.5%, 9/164) and <1 year old (8%, 2/25). Males showed higher seropositivity (8%, 6/75) than females (6.8%, 18/276). For weight, buffaloes < 100 kg had the highest seropositivity (11.1%, 1/9), followed by those >300 kg (7.4%, 17/230) and 100–300 kg (5.8%, 6/103). In adult females, seropositivity was higher in aborted buffaloes (19%, 4/21) than repeat breeders (10.5%, 4/38). However, differences in sex, weight, and reproductive problems were not statistically significant (*p* > 0.05) (Table 1).

### 3.2. Environmental Risk Factors of T. gondii in Buffaloes from Sohag

Seropositivity varied with the location of the buffaloes: The highest seropositivity was found in Sohag city, at 18.2% (2/11), followed by Akhmium, at 7.3% (20/273), Saquelta, at 4.5% (1/22), and Dar-Elsalam, at 3.8% (1/26), but differences were non-significant (*p* > 0.05). Seroprevalence of *T. gondii* varied by season: 10.7% (11/103) in spring, 5.6% (7/125) in summer, 2.2% (2/89) in winter, and 8% (2/25) in autumn, with a statistically significant difference between spring and winter (*p* = 0.022). Seropositivity was slightly higher in buffaloes with contact with cats and dogs (7.4%, 5/68) compared to those without contact (6.9%, 19/274). Additionally, smallholder management had a higher seroprevalence (10.8%, 4/37) compared to individual management (6.6%, 20/305). However, differences in contact with cats and dogs and management systems were not statistically significant (*p* > 0.05) (Table 2).

## 4. Discussion

Buffaloes act as reservoirs for many pathogens potentially affecting human health, environmental contamination, and food safety. Despite buffalo being considered a relatively resistant host, natural *T. gondii* infections have been reported [10]. This study aimed to determine the seroprevalence of *T. gondii* in water buffaloes from Sohag, southern Egypt, as a crucial step for developing prevention and control strategies in water buffaloes, a key source of milk and meat. Serological tests, particularly for IgG antibodies, are commonly used to diagnose toxoplasmosis in buffalo, as IgM persists only for a few weeks [19]. The iELISA test used in this study is highly specific, with minimal cross-reactivity, as confirmed by the manufacturer (ID. Vet, France), and is an effective diagnostic tool for various animals [20]. Our results corroborated the endemicity of *T. gondii* in Sohag using high numbers of water buffaloes for the first time. Season was identified as a predisposing factor of infection. As buffaloes are mainly infected by oocysts present in their food or in water, the observed seroprevalence indicates environmental contamination. Thus, humans are also at risk of infection through oocysts present on produce or in water, in addition to the potential infection risk when consuming raw milk or raw/undercooked meat of seropositive buffaloes. Pregnant women and immunocompromised individuals should especially try to avoid infection.

The *T. gondii* prevalence in water buffaloes was 7% (24/342). The global prevalence varies due to different parasite genotypes in different regions [21]. Our findings fall within the global range of 0–87.79% [10], aligning with studies from China (7.5%) [22], Iraq (7.4%) [23], and from Zimbabwe (5.6%) [24]. However, it was higher than studies in Brazil (1.1%) [25], India (2.9%) [26], and in the Philippines (1.9%) [27], but lower than those in Argentina (25.4%) [28], Italy (13.7%) [7], and in Mexico (30.7%) [29]. When comparing our results with studies from Egypt, our seroprevalence was similar to that reported by Selim et al. [17] but lower than studies by Farag et al. (58.6%) [14], Ibrahim et al. (8.2%) [30], Hassanain et al. (34.4%) [13], Kuraa and Malek (74.5%) [15], El-Fadaly et al. (17.1%) [31], and Ibrahim et al. (16.82%) [32]. It is higher than the rates reported by Rifaat et al. (5%) [33], and Hussein et al. (3%) [8].

We assessed various animal and environmental factors influencing *T. gondii* seropositivity in buffaloes. While buffaloes are generally resistant to toxoplasmosis [34], risk factors include the presence of cats, farm hygiene, and age [10]. Cats are key in the transmission and prevalence of *T. gondii*, shedding large numbers of oocysts in their feces [35]. In Egypt, the *T. gondii* prevalence in domestic cats reaches up to 97%, especially in rural areas, suggesting high environmental contamination with oocysts, putting not only livestock but also humans at risk of infection [36,37]. The high seroprevalence in Egypt is linked to this factor. Additionally, *T. gondii* prevalence is higher in humid, rain-prone areas, which favor oocyst sporulation [38].

Our analysis showed no significant differences in *T. gondii* seropositivity based on age, sex, weight, or reproductive issues. Seroprevalence typically increases with age, as older animals are more likely to have prolonged exposure to the parasite [39]. Female buffaloes, often older due to reproductive and milk production management, generally show higher seropositivity than males [40], though no significant difference was found in this study, consistent with other reports [41,42].

For body weight, seropositivity was 7.4% in buffaloes >300 kg and 11.1% in buffaloes <100 kg, both higher than in the 100–300 kg group (5.8%), but the differences were not significant. Older buffaloes (>300 kg) may have a higher infection risk due to longer exposure, while the higher seropositivity in lighter buffaloes (<100 kg), typically younger animals, may suggest vertical transmission. However, the lack of *T. gondii* in aborted fetuses limits the epidemiological significance of these findings [35,43].

Regarding reproductive problems, seropositivity was higher in buffaloes with a history of abortion (19%) compared to repeat breeders (10.5%), though the difference was not statistically significant. This aligns with the findings of Canada et al. [44] and Alvarado-Esquivel et al. [41]. While *T. gondii* is a known cause of abortion in other livestock, its link to abortion in buffaloes remains unclear, though some studies suggest it may cause reproductive issues like embryonic death and extended calving-to-conception intervals [41].

Seropositivity in our tested cities ranged from 3.8% to 18.2%, with no statistically significant differences. While location often affects *T. gondii* seroprevalence in bovines [12,45,46], the lack of significant differences in this study may be due to the proximity of the cities, similar management practices, and also the low sample sizes in some cities.

The seroprevalence of *T. gondii* was highest in spring (10.7%, *p* = 0.022), likely due to increased daylight, warmer temperatures, and more active behavior due to the breeding season in cats, leading to greater exposure to infections [47]. This study contrasts with others that found higher seroprevalence in summer [48] or winter [23].

Regarding contact with cats and dogs, seropositivity was similar in buffaloes with (7.4%) and without (6.9%) such contact, contrary to expectations, as cats are the definitive host of *T. gondii* [49]. In Egypt, cats have a high *T. gondii* prevalence [50], and dogs can act as mechanical carriers [51]. This result may be influenced by common management practices in Sohag, where dogs are used as guards, but the lack of seroprevalence data for cats and dogs in Sohag limits our understanding.

In Sohag, farmers generally keep female calves of buffaloes, even if they appear weak, which may lead to increased probability of the presence of diseases. Infected females may also maintain the infection via vertical transmission (no need for repeated cat contact). Furthermore, female buffaloes are more exposed to stress factors such as reproduction and milk production than male buffaloes. This may increase the probability of recurrent infections. All these factors might be responsible for the endemicity of *T. gondii* infection in buffaloes.

For management type, seroprevalence was higher in smallholder (10.8%) than individual management systems (6.6%), though the difference was not significant. These findings align with studies by Fereig et al. [12] in Qena and Metwally et al. [52] in Beheira, northern Egypt.

Regarding the limitations of this study, it would be beneficial to investigate the seroprevalence of *T. gondii* among cats and dogs present on the farms. Extending the investigation to more cities and regions from the Sohag governorate and including a higher number of buffaloes should be addressed in further studies. Such aspects would greatly help to gain more inclusive data and allow better interpretation of the results.

Our epidemiological data can serve as the foundational evidence for risk assessments of *T. gondii* infection in the tested animals and regions, allowing public health officials and organizations to transition from reactive measures to proactive, evidence-based risk mitigation. By analyzing the distribution and predisposing factors for the infection, our epidemiological data allows for the identification of high-risk populations, transmission pathways, and the effectiveness of potential interventions. Our study highlights the need to test raw milk or dairy products to assess whether viable *T. gondii* is present, and if so, to implement surveillance strategies for milk supply chains. Furthermore, the risk of transmission through consumption practices (e.g., raw or undercooked products) should be evaluated. In the meantime, public health authorities should advise farmers and consumers to pasteurize milk and to cook meat well in order to minimize the risk of infection through buffalo products.

In conclusion, toxoplasmosis in buffaloes represents an important One Health concern due to its combined economic, environmental, and public health implications. This study provides foundational epidemiological data on the presence and seasonal distribution of *T. gondii* in water buffaloes in Sohag, southern Egypt, highlighting spring as a period of elevated risk. These findings contribute to regional, data-driven surveillance efforts and underscore the value of buffaloes as sentinel animals for environmental contamination. Given the limited baseline information available for this region, the identified patterns can support future risk assessment and guide targeted control strategies. In the absence of effective vaccines, early detection, improved farm hygiene, and strengthened monitoring systems remain essential components of disease management. Expanded serological and molecular surveys involving larger sample sizes and multiple animal species are recommended to more accurately characterize transmission dynamics. Integrating routine testing of aborted animal cases and monitoring of definitive hosts, such as cats, would further enhance surveillance capacity. These measures, combined with improved food-safety awareness, can help reduce the potential zoonotic transmission of *T. gondii* within the broader One Health framework.

## Figures and Tables

**Table 1:** Effect of animal-related risk factors on seroprevalence of *T. gondii* in buffaloes.

Analyzed Factors	No. Tested	No. Negative (%)	No. Positive (%)	CI 95% *	OR (95% CI) ^+^	*p*-Value ^Φ^
Age Less than 1 year 1 to 3 years More than 3 years Total	25 164 153 342	23 (92) 155 (94.5) 140 (91.5) 318 (93)	2 (8) 9 (5.5) 13 (8.5) 24 (7)	1.4–27.5 2.7–10.5 4.8–14.4 4.6–10.4	Ref 0.7 (0.1–3.3) 1.1 (0.2–5) -	Ref 0.642 1.000 -
Sex Male Female Total	75 267 342	69 (92) 249 (93.2) 318 (93)	6 (8) 18 (6.8) 24 (7)	2.5–15.5 4.2–10.6 4.6–10.4	Ref 0.8 (0.3–2.2) -	Ref 0.798 -
Weight Less than 100 kg 100 to 300 kg More than 300 kg Total	9 103 230 342	8 (88.9) 97 (94.2) 213 (92.6) 318 (93)	1 (11.1) 6 (5.8) 17 (7.4) 24 (7)	0.6–49.3 2.4–12.8 4.5–11.8 4.6–10.4	Ref 0.5 (0.1–4.6) 0.6 (0.1–5.4) -	Ref 0.453 0.512 -
Reproductive problems Abortion Repeat breeder ^£^ Anestrus ^#^ Total	21 38 19 78	17 (81.0) 34 (89.5) 19 (100) 70 (89.7)	4 (19) 4 (10.5) 0 (0) 8 (10.3)	6.3–42.6 3.4–25.8 0–20.9 4.6–10.4	10 (0.5–200) 5.1 (0.3–99.6) Ref -	0.108 0.289 Ref -

(*) 95% CI calculated according to the method described by http://vassarstats.net/ (accessed on 22 September, 2025). (+) Odds ratio at 95% confidence interval as calculated by http://vassarstats.net/ and GraphPad Prism version 5. (Φ) *p* value was evaluated by Fisher’s exact probability test (two-tailed) by http://vassarstats.net/ and GraphPad Prism version 5. (Ref) value used as a reference. (£) refers to repeated failure of cows to conceive despite having a normal estrus cycle. (#) refers to failure of cows to show the external signs of estrus despite elapsing 3 months or more after parturition.

**Table 2:** Effect of environmental factors on seroprevalence of *T. gondii* in buffaloes.

Analyzed Factors	No. of Tested	No. of Negative (%)	No. of Positive (%)	CI 95% *	OR (95% CI) ^+^	*p*-Value ^Φ^
Location Akhmium Saquelta Dar-Elsalam Sohag El-Monsha Total	273 22 26 11 10 342	253 (92.7) 21 (95.5) 25 (96.2) 9 (81.8) 10 (100) 318 (93)	20 (7.3) 1 (4.5) 1 (3.8) 2 (18.2) 0 (0) 24 (7)	4.7–11.3 0.2–24.9 0.2–21.6 3.2–52.2 0–34.5 4.6–10.4	1.7 (0.1–30) 1.5 (0.1–39.1) 1.2 (0.1–32.9) 5.5 (0.2–130.4) Ref -	1.000 1.000 1.000 0.476 Ref -
Season Spring Summer Autumn Winter Total	103 125 25 89 342	92 (89.3) 118 (94.4) 23 (92) 87 (97.8) 318 (93)	11 (10.7) 7 (5.6) 2 (8) 2 (2.2) 24 (7)	5.7–18.7 2.5–11.6 1.4–27.5 0.4–8.7 4.6–10.4	5.2 (1.1–24.2) 2.3 (0.5–12.7) 3.8 (0.5–28.3) Ref -	0.022 0.311 0.209 Ref -
Contact to dogs and cats Yes No Total	68 274 342	63 (92.6) 255 (93.1) 318 (93)	5 (7.4) 19 (6.9) 24 (7)	2.7–17 4.3–10.8 4.6–10.4	1.1 (0.4–3) Ref -	1.000 Ref -
Management Small holders Individual Total	37 305 342	33 (89.2) 285 (93.4) 318 (93)	4 (10.8) 20 (6.6) 24 (7)	3.5–26.4 4.2–10.1 4.6–10.4	Ref 0.6 (0.2–1.8) -	Ref 0.311 -

(*) 95% CI calculated according to the method described by http://vassarstats.net/ (accessed on 22 September, 2025). (+) Odds ratio at 95% confidence interval as calculated by http://vassarstats.net/ and GraphPad Prism version 5. (Φ) *p* value was evaluated by Fisher’s exact probability test (two-tailed) by http://vassarstats.net/ and GraphPad Prism version 5. (Ref) value used as a reference.

## Data Availability

The original contributions presented in this study are included in the article. Further inquiries can be directed to the corresponding author.
